# (1*S* *R*,3*S* *R*)-[(*SR*)-Cyano­(3-phenoxy­phen­yl)meth­yl] 3-[(*Z*)-2-chloro-3,3,3-trifluoro­prop-1-en­yl]-2,2-dimethyl­cyclo­propane-1-carboxyl­ate

**DOI:** 10.1107/S1600536808034855

**Published:** 2008-11-08

**Authors:** Jing Cheng, Guangxiu Ju, Jinfeng Dong

**Affiliations:** a College of Chemistry and Molecular Sciences, Wuhan University, Wuhan 430072, People’s Republic of China

## Abstract

In the crystal of the title compound, C_23_H_19_ClF_3_NO_3_, mol­ecules are linked by one C—H⋯O hydrogen bond and two C—H⋯π inter­actions into a three-dimensional network.

## Related literature

For the insecticidal activity of the title compound, see: Anadón *et al.* (2006 ▶). For the synthesis, see: Whittle (1991 ▶).
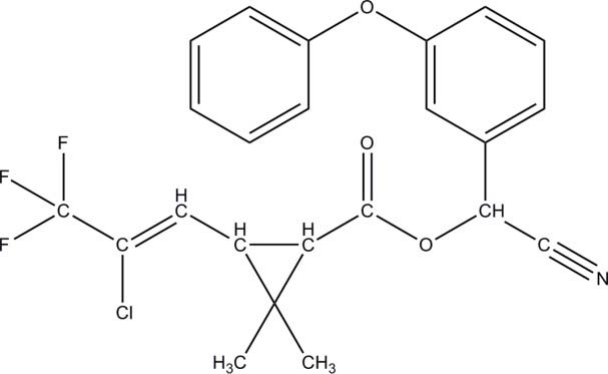


## Experimental

### 

#### Crystal data

C_23_H_19_ClF_3_NO_3_
*M* *_r_* = 449.84
Monoclinic, 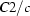

*a* = 34.7685 (13) Å
*b* = 7.0159 (3) Å
*c* = 18.6075 (7) Å
β = 102.113 (1)°
*V* = 4437.9 (3) Å^3^
*Z* = 8
Mo *K*α radiation
μ = 0.22 mm^−1^
*T* = 298 (2) K
0.30 × 0.20 × 0.20 mm

#### Data collection

Bruker SMART APEX CCD area-detector diffractometer
Absorption correction: multi-scan (*SADABS*; Bruker, 2001 ▶) *T*_min_ = 0.927, *T*_max_ = 0.957
18537 measured reflections
4361 independent reflections
2721 reflections with *I* > 2σ(*I*)
*R*_int_ = 0.040

#### Refinement

*R*[*F* ^2^ > 2σ(*F* ^2^)] = 0.055
*wR*(*F* ^2^) = 0.174
*S* = 1.03
4361 reflections
282 parameters
H-atom parameters constrained
Δρ_max_ = 0.31 e Å^−3^
Δρ_min_ = −0.31 e Å^−3^

### 

Data collection: *SMART* (Bruker, 2001 ▶); cell refinement: *SAINT-Plus* (Bruker, 2001 ▶); data reduction: *SAINT-Plus*; program(s) used to solve structure: *SHELXS97* (Sheldrick, 2008 ▶); program(s) used to refine structure: *SHELXL97* (Sheldrick, 2008 ▶); molecular graphics: *PLATON* (Spek, 2003 ▶); software used to prepare material for publication: *PLATON*.

## Supplementary Material

Crystal structure: contains datablocks global, I. DOI: 10.1107/S1600536808034855/cs2097sup1.cif

Structure factors: contains datablocks I. DOI: 10.1107/S1600536808034855/cs2097Isup2.hkl

Additional supplementary materials:  crystallographic information; 3D view; checkCIF report

## Figures and Tables

**Table 1:** Hydrogen-bond geometry (Å, °)

*D*—H⋯*A*	*D*—H	H⋯*A*	*D*⋯*A*	*D*—H⋯*A*
C13—H13⋯O3^i^	0.98	2.39	3.218 (3)	142
C21—H21⋯O3	0.93	2.34	2.984 (3)	126
C19—H19*C*⋯O3	0.96	2.39	3.037 (3)	124
C5—H5⋯*Cg*1^ii^	0.93	2.94	3.721 (1)	143 (1)
C9—H9⋯*Cg*1^iii^	0.93	3.06	3.768 (1)	134 (1)

Symmetry codes: (i) 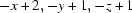; (ii) 
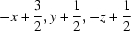
; (iii) 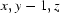. *Cg*1 is the centroid of the C1–C6 ring.

**Fig. 1.**
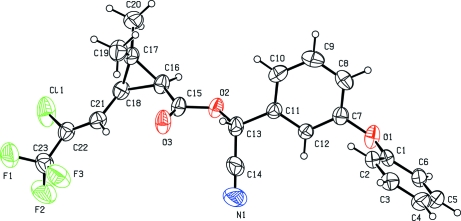
Molecular structure of the title compound showing the atom-labelling scheme. Displacement ellipsoids are drawn at 30% probability level and H atoms are shown as small spheres of arbitrary radii.

**Fig. 2.**
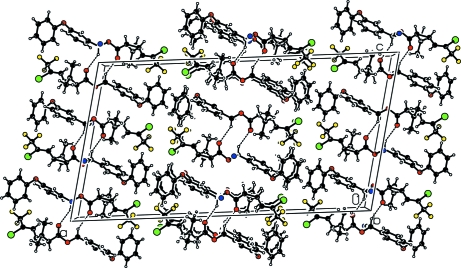
Part of the crystal structure showing the short C—H···O distances as dashed lines.

## supplementary crystallographic information

### Comment

The title compound, also known as λ-cyhalothrin, exhibits wide-spectrum insecticidal activitiy and represents a good compromise between efficacy and toxicity [Anadón *et al.*, 2006; Whittle, 1991]. In order to research into the relationship between structure and activity, we re-synthesized the title compound and report its crystal structure.

The two benzene rings (defined by atoms C1—C6 and C7—C12, respectively) connected *via* the O1 atom form a dihedral angle of 89.4 (1)° (Fig.1). The –CF3 group in this appears as a relative rare ordered one which may be due to a close approach of H21···F3 0.93/2.37 Å (idealized: 1.08/2.34 Å) (Table 1). No other unusual features are worth of mention.

In the crystal packing, the molecules at (*x*, *y*, *z*) and (-*x* + 2, -*y* + 1, *z* - 1) are linked together by C13—H13···O3^i^ interactions (Table 1), forming a dimer. Further analysis (Spek, 2003) indicates that these dimers are joined by C—H···π interactions, forming a three-dimensional network (Fig.2). In more detail, one C—H···π interaction is C5—H5···*Cg*1^ii^ [C5···*Cg*1=3.721 (1) Å, C5—H5···*Cg*1=142.8 (1)°, symmetry code: (ii) 3/2 - *x*, 1/2 + *y*, 1/2 - *z, Cg1 *is the centroid defined by phenyl C atoms C1 to C6] and the other one is C9—H9···*Cg*1^iii^(C9···*Cg*1=3.768 (1) Å, C9—H9···*Cg*1=134.0 (1)°, symmetry code: (iii) *x*, *y* - 1,*z*].

### Experimental

The title compound was synthesized according to a standard procedure (Whittle, 1991). Light blue crystals appropriate for data collection were obtained by slow evaporation of a 2-propanol solution at room temperature.

### Refinement

All the H atoms were placed at idealized geometry positions with C—H=0.93Å (aromatic), 0.98 Å (methyne) and 0.96Å (methyl), respectively. *U*_iso_ values were 1.2 times (aromatic and methyne group) or 1.5 times (methyl) of their carrier atoms.

### Crystal data

**Table:**

C_23_H_19_ClF_3_NO_3_	*F*_000_ = 1856
*M**_r_* = 449.84	*D*_x_ = 1.347 Mg m^−^^3^
Monoclinic, *C*2/*c*	Mo *K*α radiation λ = 0.71073 Å
Hall symbol: -C 2yc	Cell parameters from 4557 reflections
*a* = 34.7685 (13) Å	θ = 2.2–23.4º
*b* = 7.0159 (3) Å	µ = 0.22 mm^−^^1^
*c* = 18.6075 (7) Å	*T* = 298 (2) K
β = 102.113 (1)º	Block, light blue
*V* = 4437.9 (3) Å^3^	0.30 × 0.20 × 0.20 mm
*Z* = 8	

### Data collection

**Table:**

Bruker SMART APEX CCD area-detector diffractometer	4361 independent reflections
Radiation source: fine focus sealed Siemens Mo tube	2721 reflections with *I* > 2σ(*I*)
Monochromator: graphite	*R*_int_ = 0.040
*T* = 298(2) K	θ_max_ = 26.0º
0.3° wide ω exposures scans	θ_min_ = 2.2º
Absorption correction: multi-scan(SADABS; Bruker, 2001)	*h* = −42→35
*T*_min_ = 0.927, *T*_max_ = 0.957	*k* = −8→8
18537 measured reflections	*l* = −22→22

### Refinement

**Table:**

Refinement on *F*^2^	Secondary atom site location: difference Fourier map
Least-squares matrix: full	Hydrogen site location: inferred from neighbouring sites
*R*[*F*^2^ > 2σ(*F*^2^)] = 0.055	H-atom parameters constrained
*wR*(*F*^2^) = 0.174	*w* = 1/[σ^2^(*F*_o_^2^) + (0.1013*P*)^2^] where *P* = (*F*_o_^2^ + 2*F*_c_^2^)/3
*S* = 1.03	(Δ/σ)_max_ = 0.001
4361 reflections	Δρ_max_ = 0.31 e Å^−^^3^
282 parameters	Δρ_min_ = −0.31 e Å^−^^3^
Primary atom site location: structure-invariant direct methods	Extinction correction: none

### Fractional atomic coordinates and isotropic or equivalent isotropic displacement parameters (Å^2^)

**Table:**

	*x*	*y*	*z*	*U*_iso_*/*U*_eq_	
C1	0.82508 (6)	0.7813 (3)	0.26163 (12)	0.0597 (5)	
C2	0.82592 (6)	0.7796 (3)	0.33556 (12)	0.0629 (6)	
H2	0.8430	0.6989	0.3667	0.076*	
C3	0.80102 (8)	0.8999 (4)	0.36309 (14)	0.0784 (7)	
H3	0.8010	0.8989	0.4131	0.094*	
C4	0.77628 (9)	1.0207 (4)	0.31715 (18)	0.0913 (8)	
H4	0.7599	1.1027	0.3361	0.110*	
C5	0.77568 (8)	1.0206 (4)	0.24306 (17)	0.0874 (8)	
H5	0.7586	1.1016	0.2120	0.105*	
C6	0.80008 (7)	0.9018 (3)	0.21453 (13)	0.0737 (7)	
H6	0.7998	0.9022	0.1645	0.088*	
C7	0.87410 (7)	0.5401 (3)	0.27252 (10)	0.0620 (6)	
C8	0.86173 (8)	0.3602 (4)	0.28285 (13)	0.0772 (7)	
H8	0.8359	0.3238	0.2632	0.093*	
C9	0.88769 (10)	0.2333 (4)	0.32258 (16)	0.0874 (8)	
H9	0.8797	0.1089	0.3286	0.105*	
C10	0.92547 (9)	0.2877 (3)	0.35373 (13)	0.0758 (7)	
H10	0.9427	0.2007	0.3811	0.091*	
C11	0.93796 (6)	0.4725 (3)	0.34439 (11)	0.0573 (5)	
C12	0.91198 (6)	0.5985 (3)	0.30277 (10)	0.0559 (5)	
H12	0.9199	0.7221	0.2951	0.067*	
C13	0.97833 (6)	0.5304 (3)	0.38318 (12)	0.0702 (6)	
H13	0.9814	0.4949	0.4350	0.084*	
C14	0.98580 (8)	0.7324 (5)	0.3802 (2)	0.1129 (12)	
C15	1.04198 (6)	0.3962 (3)	0.40121 (11)	0.0623 (6)	
C16	1.06771 (6)	0.2730 (3)	0.36835 (11)	0.0636 (6)	
H16	1.0583	0.2473	0.3158	0.076*	
C17	1.09040 (6)	0.1102 (3)	0.41174 (12)	0.0649 (6)	
C18	1.11249 (7)	0.2809 (3)	0.39356 (12)	0.0645 (6)	
H18	1.1265	0.2579	0.3539	0.077*	
C19	1.08716 (8)	0.0740 (4)	0.48990 (14)	0.0892 (8)	
H19A	1.1095	0.0016	0.5145	0.134*	
H19B	1.0635	0.0040	0.4904	0.134*	
H19C	1.0864	0.1935	0.5147	0.134*	
C20	1.09345 (9)	−0.0686 (4)	0.36638 (17)	0.0966 (9)	
H20A	1.0969	−0.0324	0.3184	0.145*	
H20B	1.0698	−0.1424	0.3619	0.145*	
H20C	1.1156	−0.1434	0.3904	0.145*	
C21	1.13247 (7)	0.4171 (3)	0.44884 (12)	0.0647 (6)	
H21	1.1173	0.4799	0.4770	0.078*	
C22	1.16996 (7)	0.4567 (3)	0.46149 (12)	0.0679 (6)	
C23	1.18909 (8)	0.6047 (4)	0.51402 (15)	0.0848 (8)	
Cl1	1.20240 (2)	0.34527 (17)	0.41744 (5)	0.1270 (4)	
F1	1.21955 (6)	0.5406 (3)	0.56238 (9)	0.1209 (6)	
F2	1.20212 (7)	0.7498 (3)	0.48102 (12)	0.1395 (8)	
F3	1.16466 (6)	0.6767 (3)	0.55237 (11)	0.1256 (7)	
N1	0.99126 (9)	0.8901 (5)	0.3787 (3)	0.183 (2)	
O1	0.84875 (5)	0.6664 (3)	0.22854 (8)	0.0822 (5)	
O2	1.00732 (4)	0.4254 (2)	0.35359 (7)	0.0663 (4)	
O3	1.04889 (5)	0.4637 (3)	0.46189 (8)	0.0848 (5)	

### Atomic displacement parameters (Å^2^)

**Table:**

	*U*^11^	*U*^22^	*U*^33^	*U*^12^	*U*^13^	*U*^23^
C1	0.0391 (11)	0.0637 (13)	0.0713 (13)	−0.0075 (10)	0.0002 (10)	0.0006 (10)
C2	0.0509 (13)	0.0650 (13)	0.0686 (13)	−0.0005 (11)	0.0028 (10)	0.0014 (10)
C3	0.0761 (18)	0.0697 (15)	0.0884 (16)	0.0005 (13)	0.0146 (14)	−0.0031 (13)
C4	0.0773 (19)	0.0685 (16)	0.128 (2)	0.0105 (14)	0.0213 (17)	−0.0024 (17)
C5	0.0674 (17)	0.0660 (16)	0.119 (2)	0.0060 (13)	−0.0019 (15)	0.0178 (15)
C6	0.0554 (14)	0.0761 (15)	0.0808 (14)	−0.0074 (13)	−0.0056 (12)	0.0131 (13)
C7	0.0586 (14)	0.0718 (15)	0.0553 (11)	0.0046 (11)	0.0112 (10)	−0.0011 (10)
C8	0.0673 (16)	0.0902 (19)	0.0738 (14)	−0.0221 (15)	0.0141 (12)	−0.0038 (13)
C9	0.108 (2)	0.0645 (16)	0.0941 (18)	−0.0241 (16)	0.0301 (17)	−0.0003 (14)
C10	0.0889 (19)	0.0599 (15)	0.0778 (14)	0.0141 (14)	0.0157 (14)	0.0110 (12)
C11	0.0534 (13)	0.0582 (13)	0.0603 (11)	0.0078 (10)	0.0123 (9)	−0.0030 (10)
C12	0.0518 (13)	0.0520 (11)	0.0639 (11)	0.0010 (10)	0.0120 (10)	0.0011 (9)
C13	0.0504 (14)	0.0838 (17)	0.0741 (13)	0.0169 (12)	0.0080 (11)	−0.0148 (12)
C14	0.0446 (15)	0.089 (2)	0.198 (4)	0.0039 (15)	0.0085 (18)	−0.043 (2)
C15	0.0502 (13)	0.0796 (15)	0.0567 (12)	0.0140 (11)	0.0105 (10)	0.0005 (11)
C16	0.0570 (14)	0.0797 (15)	0.0551 (11)	0.0162 (11)	0.0140 (10)	−0.0037 (10)
C17	0.0475 (12)	0.0663 (14)	0.0805 (14)	0.0074 (11)	0.0129 (10)	0.0040 (11)
C18	0.0540 (13)	0.0745 (15)	0.0702 (13)	0.0096 (11)	0.0245 (10)	−0.0003 (11)
C19	0.0715 (17)	0.100 (2)	0.0950 (17)	−0.0010 (14)	0.0155 (13)	0.0306 (15)
C20	0.0759 (19)	0.0710 (16)	0.141 (2)	0.0087 (14)	0.0185 (17)	−0.0167 (16)
C21	0.0573 (15)	0.0660 (14)	0.0759 (13)	0.0057 (11)	0.0256 (11)	0.0025 (11)
C22	0.0564 (15)	0.0767 (15)	0.0733 (13)	0.0060 (12)	0.0194 (11)	0.0116 (12)
C23	0.0679 (18)	0.0958 (19)	0.0901 (17)	−0.0137 (15)	0.0157 (15)	0.0084 (16)
Cl1	0.0578 (5)	0.2056 (10)	0.1202 (6)	0.0220 (5)	0.0247 (4)	−0.0386 (6)
F1	0.0990 (13)	0.1413 (15)	0.1050 (12)	−0.0063 (11)	−0.0183 (10)	0.0062 (11)
F2	0.1411 (17)	0.1298 (15)	0.1407 (15)	−0.0612 (13)	0.0139 (13)	0.0315 (12)
F3	0.1030 (14)	0.1286 (15)	0.1494 (15)	−0.0232 (11)	0.0360 (12)	−0.0558 (12)
N1	0.075 (2)	0.086 (2)	0.377 (6)	−0.0026 (17)	0.022 (3)	−0.054 (3)
O1	0.0645 (10)	0.1168 (14)	0.0603 (9)	0.0232 (10)	0.0019 (8)	0.0091 (9)
O2	0.0542 (9)	0.0836 (11)	0.0590 (8)	0.0222 (8)	0.0071 (7)	−0.0039 (7)
O3	0.0577 (10)	0.1282 (14)	0.0655 (9)	0.0242 (9)	0.0065 (7)	−0.0257 (9)

### Geometric parameters (Å, °)

**Table:**

C1—C2	1.370 (3)	C13—H13	0.9800
C1—C6	1.385 (3)	C14—N1	1.124 (4)
C1—O1	1.386 (3)	C15—O3	1.201 (2)
C2—C3	1.382 (3)	C15—O2	1.353 (2)
C2—H2	0.9300	C15—C16	1.467 (3)
C3—C4	1.371 (4)	C16—C17	1.521 (3)
C3—H3	0.9300	C16—C18	1.530 (3)
C4—C5	1.374 (4)	C16—H16	0.9800
C4—H4	0.9300	C17—C18	1.499 (3)
C5—C6	1.373 (4)	C17—C19	1.504 (3)
C5—H5	0.9300	C17—C20	1.528 (3)
C6—H6	0.9300	C18—C21	1.468 (3)
C7—C8	1.360 (3)	C18—H18	0.9800
C7—C12	1.381 (3)	C19—H19A	0.9600
C7—O1	1.389 (3)	C19—H19B	0.9600
C8—C9	1.369 (4)	C19—H19C	0.9600
C8—H8	0.9300	C20—H20A	0.9600
C9—C10	1.374 (4)	C20—H20B	0.9600
C9—H9	0.9300	C20—H20C	0.9600
C10—C11	1.390 (3)	C21—C22	1.305 (3)
C10—H10	0.9300	C21—H21	0.9300
C11—C12	1.379 (3)	C22—C23	1.485 (4)
C11—C13	1.494 (3)	C22—Cl1	1.716 (2)
C12—H12	0.9300	C23—F1	1.317 (3)
C13—C14	1.444 (4)	C23—F2	1.317 (3)
C13—O2	1.448 (2)	C23—F3	1.319 (3)
			
C2—C1—C6	121.3 (2)	O2—C15—C16	110.69 (17)
C2—C1—O1	123.49 (19)	C15—C16—C17	121.01 (18)
C6—C1—O1	115.2 (2)	C15—C16—C18	121.43 (18)
C1—C2—C3	118.9 (2)	C17—C16—C18	58.87 (14)
C1—C2—H2	120.5	C15—C16—H16	114.7
C3—C2—H2	120.5	C17—C16—H16	114.7
C4—C3—C2	120.4 (2)	C18—C16—H16	114.7
C4—C3—H3	119.8	C18—C17—C19	119.9 (2)
C2—C3—H3	119.8	C18—C17—C16	60.88 (14)
C3—C4—C5	120.0 (3)	C19—C17—C16	120.3 (2)
C3—C4—H4	120.0	C18—C17—C20	115.7 (2)
C5—C4—H4	120.0	C19—C17—C20	115.1 (2)
C6—C5—C4	120.5 (2)	C16—C17—C20	114.3 (2)
C6—C5—H5	119.7	C21—C18—C17	123.46 (18)
C4—C5—H5	119.7	C21—C18—C16	122.67 (18)
C5—C6—C1	118.8 (2)	C17—C18—C16	60.26 (15)
C5—C6—H6	120.6	C21—C18—H18	113.5
C1—C6—H6	120.6	C17—C18—H18	113.5
C8—C7—C12	121.4 (2)	C16—C18—H18	113.5
C8—C7—O1	119.6 (2)	C17—C19—H19A	109.5
C12—C7—O1	119.0 (2)	C17—C19—H19B	109.5
C7—C8—C9	119.2 (2)	H19A—C19—H19B	109.5
C7—C8—H8	120.4	C17—C19—H19C	109.5
C9—C8—H8	120.4	H19A—C19—H19C	109.5
C8—C9—C10	120.7 (2)	H19B—C19—H19C	109.5
C8—C9—H9	119.6	C17—C20—H20A	109.5
C10—C9—H9	119.6	C17—C20—H20B	109.5
C9—C10—C11	120.2 (2)	H20A—C20—H20B	109.5
C9—C10—H10	119.9	C17—C20—H20C	109.5
C11—C10—H10	119.9	H20A—C20—H20C	109.5
C12—C11—C10	118.9 (2)	H20B—C20—H20C	109.5
C12—C11—C13	122.4 (2)	C22—C21—C18	124.9 (2)
C10—C11—C13	118.7 (2)	C22—C21—H21	117.5
C11—C12—C7	119.7 (2)	C18—C21—H21	117.5
C11—C12—H12	120.2	C21—C22—C23	124.1 (2)
C7—C12—H12	120.2	C21—C22—Cl1	123.29 (19)
C14—C13—O2	109.9 (2)	C23—C22—Cl1	112.62 (18)
C14—C13—C11	114.0 (2)	F1—C23—F2	106.1 (2)
O2—C13—C11	109.65 (17)	F1—C23—F3	106.1 (2)
C14—C13—H13	107.7	F2—C23—F3	106.3 (3)
O2—C13—H13	107.7	F1—C23—C22	113.3 (2)
C11—C13—H13	107.7	F2—C23—C22	112.6 (2)
N1—C14—C13	179.0 (4)	F3—C23—C22	111.9 (2)
O3—C15—O2	122.07 (19)	C1—O1—C7	118.18 (16)
O3—C15—C16	127.24 (19)	C15—O2—C13	115.04 (15)
			
C6—C1—C2—C3	0.5 (3)	C15—C16—C17—C20	142.7 (2)
O1—C1—C2—C3	−179.9 (2)	C18—C16—C17—C20	−107.0 (2)
C1—C2—C3—C4	−0.9 (4)	C19—C17—C18—C21	1.4 (3)
C2—C3—C4—C5	1.0 (4)	C16—C17—C18—C21	111.6 (2)
C3—C4—C5—C6	−0.8 (4)	C20—C17—C18—C21	−143.6 (2)
C4—C5—C6—C1	0.4 (4)	C19—C17—C18—C16	−110.2 (2)
C2—C1—C6—C5	−0.3 (3)	C20—C17—C18—C16	104.8 (2)
O1—C1—C6—C5	−179.9 (2)	C15—C16—C18—C21	−3.2 (3)
C12—C7—C8—C9	−1.6 (3)	C17—C16—C18—C21	−112.8 (2)
O1—C7—C8—C9	176.0 (2)	C15—C16—C18—C17	109.7 (2)
C7—C8—C9—C10	2.0 (4)	C17—C18—C21—C22	121.1 (3)
C8—C9—C10—C11	−0.8 (4)	C16—C18—C21—C22	−165.3 (2)
C9—C10—C11—C12	−0.8 (3)	C18—C21—C22—C23	175.5 (2)
C9—C10—C11—C13	175.9 (2)	C18—C21—C22—Cl1	−3.1 (3)
C10—C11—C12—C7	1.2 (3)	C21—C22—C23—F1	127.1 (3)
C13—C11—C12—C7	−175.40 (18)	Cl1—C22—C23—F1	−54.1 (3)
C8—C7—C12—C11	0.0 (3)	C21—C22—C23—F2	−112.6 (3)
O1—C7—C12—C11	−177.66 (17)	Cl1—C22—C23—F2	66.2 (3)
C12—C11—C13—C14	6.9 (3)	C21—C22—C23—F3	7.1 (3)
C10—C11—C13—C14	−169.7 (2)	Cl1—C22—C23—F3	−174.13 (19)
C12—C11—C13—O2	−116.8 (2)	C2—C1—O1—C7	1.3 (3)
C10—C11—C13—O2	66.6 (3)	C6—C1—O1—C7	−179.1 (2)
O3—C15—C16—C17	43.6 (4)	C8—C7—O1—C1	90.7 (2)
O2—C15—C16—C17	−135.4 (2)	C12—C7—O1—C1	−91.6 (2)
O3—C15—C16—C18	−26.5 (4)	O3—C15—O2—C13	−4.7 (3)
O2—C15—C16—C18	154.5 (2)	C16—C15—O2—C13	174.40 (18)
C15—C16—C17—C18	−110.3 (2)	C14—C13—O2—C15	82.4 (3)
C15—C16—C17—C19	−0.7 (3)	C11—C13—O2—C15	−151.59 (19)
C18—C16—C17—C19	109.6 (2)		

### Hydrogen-bond geometry (Å, °)

**Table:**

*D*—H···*A*	*D*—H	H···*A*	*D*···*A*	*D*—H···*A*
C13—H13···O3^i^	0.98	2.39	3.218 (3)	142
C21—H21···O3	0.93	2.34	2.984 (3)	126
C19—H19C···O3	0.96	2.39	3.037 (3)	124
C21—H21···F3	0.93	2.37	2.716 (2)	102
C5—H5···Cg1^ii^	0.93	2.94	3.721 (1)	143 (1)
C9—H9···Cg1^iii^	0.93	?	3.768 (1)	134 (1)

Symmetry codes: (i) −*x*+2, −*y*+1, −*z*+1; (ii) −*x*+3/2, *y*+1/2, −*z*+1/2; (iii) *x*, *y*−1, *z*.
